# Association between exposure to air pollution and thalamus volume in adults: A cross-sectional study

**DOI:** 10.1371/journal.pone.0230829

**Published:** 2020-03-30

**Authors:** Dawson W. Hedges, Lance D. Erickson, Shawn D. Gale, Jacqueline E. Anderson, Bruce L. Brown

**Affiliations:** 1 Department of Psychology, Brigham Young University, Provo, Utah, United States of America; 2 The Neuroscience Center, Brigham Young University, Provo, Utah, United States of America; 3 Department of Sociology, Brigham Young University, Provo, Utah, United States of America; Telethon Institute for Child Health Research, AUSTRALIA

## Abstract

**Background:**

Air pollution has been associated with cognitive function and brain volume. While most previous research has examined the association between air pollution and brain volume in cortical structures or total brain volume, less research has investigated associations between exposure to air pollution and subcortical structures, including the thalamus. Further, the few available previous studies investigating associations between air pollution and thalamic volume have shown mixed results.

**Methods:**

In this study, we evaluated the association between PM_2.5_, PM_2.5–10_, PM_10_, nitrogen dioxide, and nitrogen oxides and volume of the thalamus in adults using the UK Biobank resource, a large community-based sample, while adjusting for multiple covariates that could confound an association between air pollution and thalamic volume.

**Results:**

In adjusted models, the left but not right thalamus volume was significantly inversely associated with PM_2.5–10_, although there were no significant associations between PM_2.5_, PM_10_, nitrogen dioxide, and nitrogen oxides with either left or right thalamic volumes. In addition, interactions between age and PM_2.5–10_ and PM_10_ were inversely associated with thalamic volume, such that thalamic volume in older people appeared more vulnerable to the adverse effects of PM_2.5–10_ and PM_10_, and interactions between educational attainment and PM_2.5_, nitrogen dioxide, and nitrogen oxides and between self-rated health and PM_2.5–10_ were positively associated with thalamic volume, such that higher educational attainment and better self-rated health appeared protective against the adverse effects of air pollution on the thalamus.

**Conclusion:**

These findings suggest a possible association between thalamic volume and air pollution particularly in older people and in people with comparatively low educational attainment at levels of air pollution found in the United Kingdom.

## Introduction

Accumulating evidence suggests that exposure to air pollution appears to adversely affect the brain [1], and air pollution has been associated with decreased cognitive function [2], dementia [3], and psychosis in adolescents [4]. Further, air pollution has been associated with smaller brain volumes, including smaller prefrontal volume in children [5] and older adults [6, 7], smaller cortical white-matter volume in older women [8] and in children [1], and cortical gray matter [9].

While the majority of studies investigating associations between air pollution and brain volume have focused on cortical areas, preliminary evidence has found possible associations with some subcortical structures. In this regard, air pollution in one study [10] but not all [6–8, 11] was associated with hippocampal volume. Air pollution also might be associated with smaller volumes of the basal ganglia [9]. There is less information, however, about the associations between air pollution and other subcortical brain structures, although one study found an association between PM_2.5_ and subcortical white matter [6]. In another of the few available studies examining relationships betweeen air pollution and subcortical structures, Power et al. [7] found an associaton between particulate matter with diameter less than or equal to 2.5 μm (PM_2.5_) and particulate matter with a diameter less than or equal to 10 μm (PM_10_) and total aggregate caudate, putamen, pallidum, and thalamus volume. In contrast, another study that investigated the association between PM_2.5_ only and thalamic volume found a positive association between PM_2.5_ and thalamic volume [6].

Because the thalamus appears vulnerable to injury [12–16] and is important in cognition [17] and based on previous findings of associations between air pollution and overall caudate, putamen, pallidum and thalamus volume, we sought to investigate further the association between measures of air pollution and thalamic volume in adults. Because prior work has suggested that factors such as age, sex, and education may modify the health effects of air pollution [18], we investigated interaction effects between several variables and air pollution. To investigate this potential association, we used available air-pollution, thalamic and total-brain volume, demographic, and medical data from the large, community-based UK Biobank Resource (http://www.ukbiobank.ac.uk).

## Methods

### Study sample

In this study, we used data from participants from the UK Biobank, which initially enrolled approximately 500,000 adults sampled from population-based registries between 2006 and 2010 (http://www.ukbiobank.ac.uk) [19]. The UK Biobank obtained demographic and medical information from participants via questionnaires, a nurse interview, and physical examinations (http://biobank.ctsu.ox.ac.uk/crystal/field.cgi?id=200). Beginning in April 2014, a subsample of the participants approximately between the ages of 44 and 80 subsequently had magnetic-resonance brain imaging (UK Biobank Brain Imaging Documentation, http://www.ukbiobank.ac.uk). At the time we received regulatory approval, there were 21,407 participants with processed MRI data made available, of whom we limited our analyses to the 18,278 participants who had data for exposure to air pollution and the chosen control variables.

The ethical approval for UK Biobank studies is from the National Research Ethics Service Committee North West–Haydock (REC Ref 11/NW/0382). All participants in the UK Biobank provided informed consent (http://biobank.ctsu.ox.ac.uk/crystal/field.cgi?id=200). We applied for access to anonymized data, and the UK Biobank gave us regulatory approval to conduct this research (UK Biobank Resource under Application Number 41535).

#### Thalamic volume

Details regarding image acquisition and processing are available on the UK Biobank website. Briefly, the UK Biobank used a 3-Tesla, 32-channel coil Siemens Skyra scanner (Siemens Medical Solutions, Germany) (biobank.ctsu.ox.ac.uk/crystal/docs/brain_mri.pdf) [20, 21] for all imaging acquisition. For our analyses, we used the pre-processed three-dimensional magnetization prepared for rapid echo-gradient (3D MP-RAGE) T1-weighted image-derived phenotypes with numerical data for left and right gray-matter volume of the thalamus and total brain white-matter and gray-matter volume normalized for head size that we obtained from the UK Biobank. The resolution was 1 x 1 x 1mm, and the field view was 208 x 256 x 256 (biobank.ctsu.ox.ac.uk/crystal/docs/brain_mri.pdf). The magnetic resonance imaging we used was obtained from 2014 to 2019.

### Air pollution measures

We used address-level estimates of exposure to PM_2.5_, PM_2.5–10_, and PM_10_ and to nitrogen dioxide (NO_2_) and nitrogen oxides (NO_X_) concentrations measured over a year (biobank.ctsu.ox.ac.uk/crystal/label.cgi?id = 114) as the independent variable. The UK Biobank used air-pollution data obtained from Small Area Health Statistics Unit (http://www.sahsu.org) in conjunction with the BioShaRE-EU Environmental Determinants of Health Project (https://biobank.ndph.ox.ac.uk/showcase/label.cgi?id=115). The air-pollution estimates we used are from 2010 land-use regression models of the European Study of Cohorts for Air Pollution Effects (http:/www.escapeproject.eu/) and Eurostreets traffic data for 2008 (biobank.ctsu.ox.ac.uk/crystal/label.cgi?id = 114). More detailed information about the particulate-matter data is available with Eeftens et al. [22] and about the NO_2_ and NO_x_ data is available at Beelen et al. [23].

### Covariates

To control for potential confounding, we adjusted the statistical models for variables that potentially could be associated with brain volume. To do so, we included available variables that have been associated with cognitive function, assuming that variables associated with cognitive function also could be associated with brain structure [24–26] and accordingly included age, sex (self-report), race-ethnicity, educational attainment measured as achieving a college degree versus less than a college degree, annual household income in pounds, self-rated health, body-mass index, smoking history, and frequency of alcohol use as covariates. We also adjusted for the inverse of the distance to the nearest major road based on the relationship between air and noise pollution [27]. We also included total gray and white matter adjusted for head size [20] to control for overall brain volume. Finally, we included the self-rated measure of overall health (excellent, good, fair, poor) as a covariate. We recoded these data so that higher scores represtented assessments of better health.

The interactions that are presented in Tables 3 through Table 6 are simply the product of the specific covariate (e.g., age in Table 3) with an air pollution measure. We did not center the variables because we were concerned with the product term (i.e., interaction effect) rather than the main effects in the interaction models.

### Statistical analysis

We used linear regression models to evaluate associations between air pollution and left and right thalamic volumes and included the identified covariates in the models to adjust for potential confounding. Furthermore, we investigated separate interaction effects between age, sex, educational attainment, and overall self-rated health in the association between each air pollutant and thalamic volume by adding an interaction term (e.g., age by PM_2.5_) to the adjusted linear-regression models. We investigated these interactions because of prior work suggests that increased susceptibility to air pollution might be associated with age [2, 28], that age may interact with modifiers of brain morphology [29], that sex differences in brain morphology exist [30] and may interact with air-pollution-related injury, and that air pollution might affect women and men differently [31]. Because education has been associated with both brain volume [32] and risk for neurodegenerative disease [33], there could potentially be an interaction with education since educational attainment appears to be protective in both cognitive aging and in response to brain pathology [34]. To investigate whether air pollution was related to thalamus volume in a non-linear way, we estimated a series of models (not shown) that included a quadratic term for the respective measures of air pollution. We carried out all statistical analyses with Stata 15.1 (StataCorp, Stata Statistical Software, Release 15. College Station, Texas).

## Results

The total number of participants was 18,278. Table 1 includes means and standard deviations for continuous variables and proportions for dichotomous or polytomous variables. The average age of the participants was 62.15 years and ranged from approximately 44 to 80 years. Ninety-seven percent of the sample were white, 52 percent were female, and 50 percent had completed college. Table 1 also reports average pollution levels for each of the available air pollution components, left and right thalamic volume, brain volume, and values of the covariates.

**Table 1:** Descriptive statistics of study variables.

	Mean or Proportion	SD	Minimum	Maximum
Thalamus volume (mm^3^)				
Left	7808.25	749.70	4209.00	14641.00
Right	7613.39	727.54	4644.00	13728.00
Total brain volume (mm^3^)	1504427.91	72550.79	1151700.00	1793910.00
Air pollution (ug/m^3^)				
Particulate matter 2.5	9.90	1.01	8.17	19.65
Particulate matter 2.5 to10	6.36	.88	5.57	10.25
Particulate matter 10	16.01	1.85	11.78	25.21
Nitrogen dioxide	25.61	6.86	12.93	89.55
Nitrogen oxides	42.32	14.02	19.74	231.89
Inverse distance to major road	.01	.01	.00	.91
Age	62.15	7.44	44.00	80.00
Female	.52		.00	1.00
Race				
White	.97		.00	1.00
Black	.01		.00	1.00
Asian	.01		.00	1.00
Other	.01		.00	1.00
College degree	.50		.00	1.00
Household income (in pounds)				
< 18k	.12		.00	1.00
18k - 30,999	.28		.00	1.00
31k - 51,999	.31		.00	1.00
52k - 100k	.23		.00	1.00
> 100k	.06		.00	1.00
Self-rated health	2.99	.66	1.00	4.00
Body-mass index	26.59	4.42	13.39	58.70
Smoking status				
Non-smoker	.63		.00	1.00
Past	.33		.00	1.00
Current	.04		.00	1.00
Drinking frequency				
Daily or almost daily	.17		.00	1.00
3–4 times/week	.28		.00	1.00
Once or twice/week	.27		.00	1.00
1–3 times/month	.12		.00	1.00
Special occasions	.10		.00	1.00
Never	.06		.00	1.00

N = 18,278. Source: UK Biobank.

The nonlinear models showed no evidence of nonlinear effects between concentrations of PM_2.5_ and PM_2.5–10_ and thalamic volumes, although at higher concentrations of PM_10_, there was evidence of slight nonlinearity. As such, because none of the quadratic terms was statistically significant, we thought that the linear models accurately captured the relationships between air pollution and thalamic volume and report only the results of the linear models.

Higher PM_2.5–10_ was associated with less left but not right thalamic volume in adjusted models (Table 2). In contrast, there were no associations between PM_2.5_, PM_10_, NO_2_, and NO_X_ and either left or right thalamic volume in adjusted models (Table 2).

**Table 2:** Thalamus volume (mm^3^) and air pollution: Unstandardized coefficients and their 95% confidence intervals and standardized coefficients from linear regression.

	Left Thalamus Volume			Right Thalamus Volume		
	b	95% CI	Beta	b	95% CI	Beta
Particulate Matter 2.5						
Unadjusted	-1.69	-12.43,9.05	-1.79	-2.74	-13.16,7.69	-2.89
Adjusted	-6.57	-15.79,2.66	-6.94	-6.39	-15.23,2.44	-6.76
Particulate Matter 2.5 to 10						
Unadjusted	-11.92	-24.21,.37	-10.74	-9.22	-21.15,2.71	-8.31
Adjusted	-12.06*	-22.39,-1.73	-10.86	-8.71	-18.60,1.19	-7.84
Particulate Matter 10						
Unadjusted	-4.95	-10.82,.92	-9.40	-3.88	-9.58,1.81	-7.37
Adjusted	-4.07	-9.02,.88	-7.73	-2.58	-7.33,2.16	-4.91
Nitrogen Dioxide						
Unadjusted	1.29	-.30,2.87	9.77	.83	-.71,2.37	6.30
Adjusted	.00	-1.35,1.36	.04	-.27	-1.57,1.02	-2.08
Nitrogen Oxides						
Unadjusted	.10	-.67,.88	1.62	-.18	-.93,.57	-2.81
Adjusted	-.31	-1.00,.37	-4.89	-.52	-1.17,.13	-8.11

Adjusted models include total brain volume, inverse distance to nearest major road, age, gender, race, education, income, overall health, BMI, smoking status and frequency of drinking alcohol. N = 18,278.

* p < .05.

Source: *UK Biobank*.

**Fig 1:**
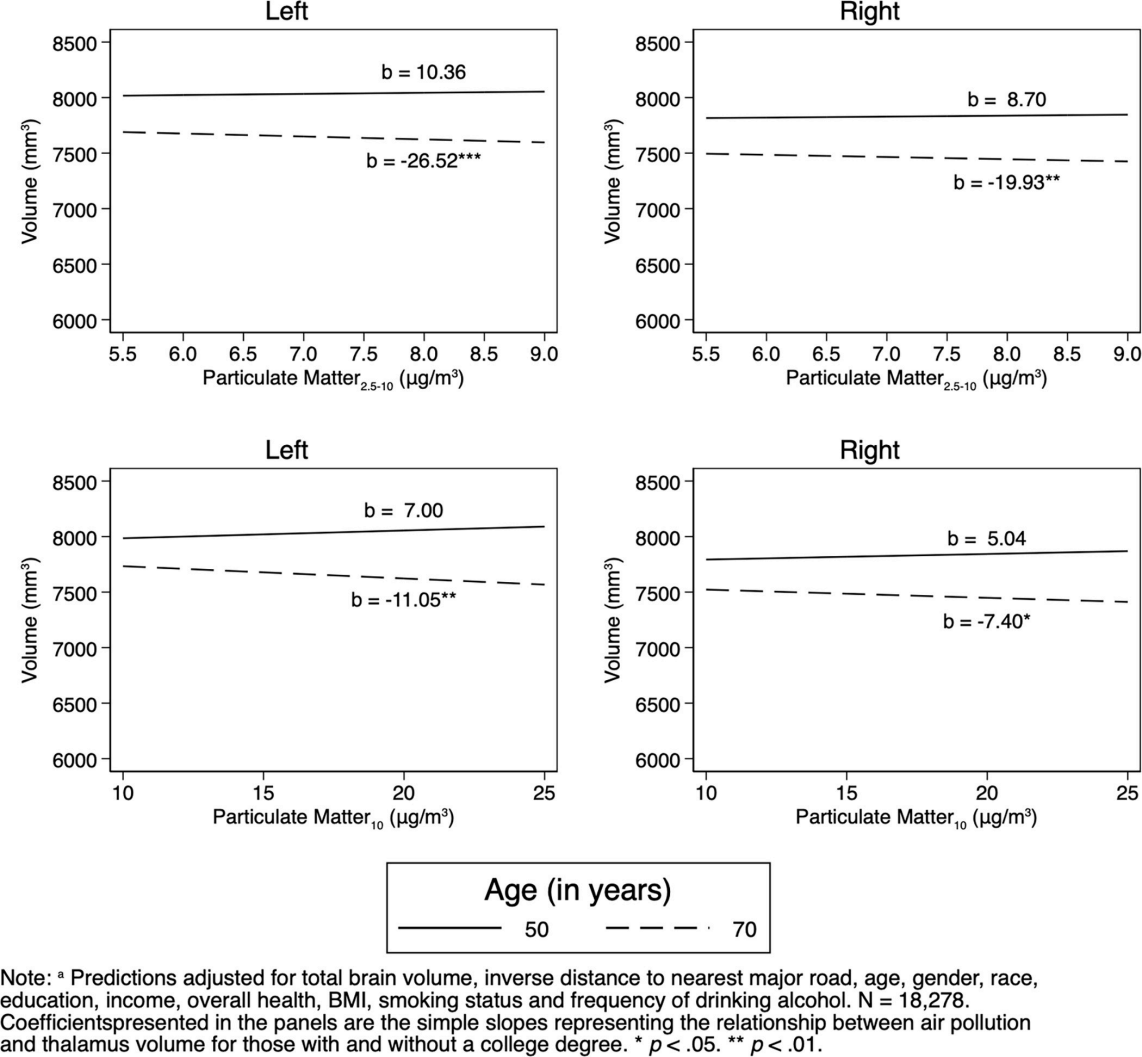
Interactions of pollution and age on thalamus volume: Adjusted predictions^a^ from OLS regressions.

**Fig 2:**
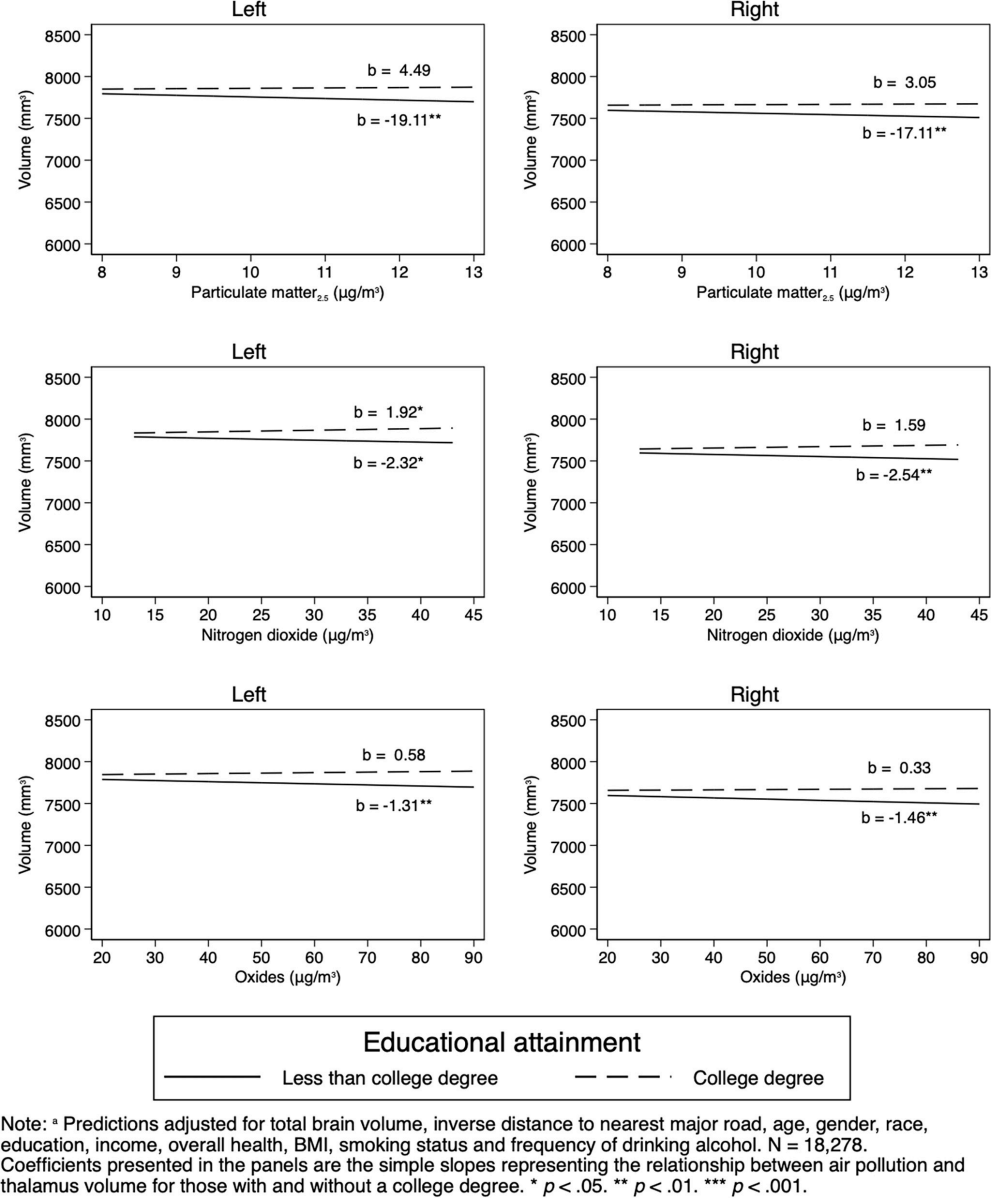
Interactions of pollution and educational attainment on thalamus volume: Adjusted predictions^a^ from OLS regressions.

However, we did find interactions between air pollutants and age: higher PM_2.5–10_ and increased age and higher PM_10_ and age interacted and were associated with less left and right thalamus volume (Table 3 and Fig 1). While did not find interactions with sex (Table 4), there were interactions between educational attainment and PM_2.5_, NO_2_, and NO_X_ (Table 5 and Fig 2) that were associated with left and right thalamic volumes, such that educational attainment appeared protective for thalamic volume against PM_2.5_, NO_2_, and NO_X_. Finally, an interaction between PM_2.5–10_ and self-rated health predicted left but not right thalamic volume such that higher self-rated health appeared protective against PM_2.5–10_ (Table 6).

**Table 3:** Thalamus volume (mm^3^) and interactions between air pollution and age: Unstandardized coefficient with 95% confidence intervals and standardized coefficients from linear regression.

	Left Thalamus Volume			Right Thalamus Volume		
	b	95% CI	Beta	b	95% CI	Beta
Particulate Matter 2.5						
Pollutant	22.10	-52.45,96.65	23.36	-5.23	-76.65,66.20	-5.52
Age	-13.50*	-25.43,-1.57	-18.12	-17.19**	-28.62,-5.76	-17.38
Interaction	-.46	-1.66,.73	-.49	-.02	-1.16,1.12	-.02
Particulate Matter 2.5 to 10						
Pollutant	102.56*	18.42,186.70	92.37	80.26	-.36,160.87	72.28
Age	-6.28	-14.96,2.41	-18.13	-8.19	-16.51,.13	-17.39
Interaction	-1.84**	-3.19,-.50	-1.66	-1.43*	-2.72,-.14	-1.29
Particulate Matter 10						
Pollutant	52.12*	11.46,92.79	98.99	36.14	-2.82,75.11	68.64
Age	-3.55	-14.07,6.96	-18.21	-7.32	-17.40,2.75	-17.42
Interaction	-.90**	-1.55,-.25	-1.71	-.62*	-1.24,-.00	-1.18
Nitrogen Dioxide						
Pollutant	-1.08	-12.08,9.91	-8.21	-4.06	-14.59,6.47	-30.75
Age	-18.44***	-23.21,-13.66	-17.97	-18.88***	-23.45,-14.30	-17.25
Interaction	.02	-.16,.19	.13	.06	-.11,.23	.46
Nitrogen Oxides						
Pollutant	-.99	-6.39,4.41	-15.43	-3.48	-8.65,1.69	-54.03
Age	-18.52***	-22.50,-14.54	-18.04	-19.42***	-23.24,-15.61	-17.32
Interaction	.01	-.08,.10	.17	.05	-.04,.13	.74

All models include total brain volume, inverse distance to nearest major road, age, gender, race, education,

Income, overall health, BMI smoking status, and frequency of drinking alcohol. N = 18,278.

* p < .05.

** p < .01,

*** p < .001.

Source: *UK Biobank*.

**Table 4:** Thalamus volume (mm^3^) and interactions between air pollution and sex: Unstandardized coefficient with 95% confidence intervals and standardized coefficients from linear regression.

	Left Thalamus Volume			Right Thalamus Volume		
	b	95% CI	Beta	b	95% CI	Beta
Particulate Matter 2.5						
Pollutant	-10.09	-22.99,2.82	-10.66	-8.10	-20.46,4.27	-8.56
Female	-747.86***	-922.92,-572.80	-679.39	-718.16***	-885.88,-550.45	-685.03
Interaction	6.85	-10.73,24.43	7.24	3.32	-13.52,20.16	3.51
Particulate Matter 2.5 to 10						
Pollutant	-9.60	-24.07,4.88	-8.64	-1.66	-15.53,12.20	-1.50
Female	-649.10***	-777.96,-520.24	-680.40	-596.73***	-720.19,-473.27	-686.28
Interaction	-4.87	-24.93,15.19	-4.39	-13.93	-33.15,5.29	-12.55
Particulate Matter 10						
Pollutant	-3.38	-10.28,3.52	-6.41	.16	-6.46,6.77	.30
Female	-657.91***	-812.35,-503.47	-680.32	-597.83***	-745.79,-449.87	-686.46
Interaction	-1.38	-10.96,8.20	-2.62	-5.46	-14.63,3.72	-10.37
Nitrogen Dioxide						
Pollutant	-.13	-2.03,1.78	-.95	-.37	-2.20,1.45	-2.84
Female	-686.17***	-755.02,-617.32	-679.42	-690.08***	-756.04,-624.12	-684.90
Interaction	.25	-2.33,2.84	1.91	.19	-2.29,2.67	1.47
Nitrogen Oxides						
Pollutant	-.26	-1.20,.69	-3.98	-.52	-1.42,.39	-8.01
Female	-675.08***	-731.84,-618.31	-680.06	-684.78***	-739.17,-630.40	-685.33
Interaction	-.11	-1.38,1.15	-1.76	-.01	-1.23,1.20	-.19

All models include total brain volume, inverse distance to nearest major road, age, gender, race, education,

Income, overall health, BMI smoking status, and frequency of drinking alcohol. N = 18,278.

* p < .05.

** p < .01,

*** p < .001.

Source: *UK Biobank*.

**Table 5:** Thalamus volume (mm^3^) and interactions between air pollution and education: Unstandardized coefficient with 95% confidence intervals and standardized coefficients from linear regression.

	Left Thalamus Volume			Right Thalamus Volume		
	b	95% CI	Beta	b	95% CI	Beta
Particulate Matter 2.5						
Pollutant	-19.11**	-32.22,-5.99	-20.20	-17.11**	-29.68,-4.54	-18.09
College degree	-132.89	-307.60,41.83	102.87	-99.03	-266.42,68.37	102.47
Interaction	23.59**	6.04,41.15	24.94	20.17*	3.35,36.99	21.32
Particulate Matter 2.5 to 10						
Pollutant	-11.55	-25.31,2.20	-10.41	-10.22	-23.40,2.96	-9.20
College degree	106.52	-22.98,236.02	99.30	78.21	-45.86,202.29	99.81
Interaction	-1.12	-21.29,19.05	-1.01	3.36	-15.96,22.68	3.03
Particulate Matter 10						
Pollutant	-7.29*	-14.08,-.51	-13.85	-6.42	-12.92,.08	-12.19
College degree	-6.74	-161.35,147.88	101.39	-26.92	-175.05,121.22	101.76
Interaction	6.66	-2.93,16.25	12.65	7.92	-1.26,17.11	15.05
Nitrogen Dioxide						
Pollutant	-2.32*	-4.28,-.36	-17.58	-2.54**	-4.42,-.66	-19.21
College degree	-8.11	-76.94,60.72	105.25	-5.04	-70.98,60.90	105.18
Interaction	4.24**	1.65,6.84	32.15	4.13**	1.64,6.61	31.26
Nitrogen Oxides						
Pollutant	-1.31**	-2.26,-.36	-20.30	-1.46**	-2.37,-.55	-22.72
College degree	20.62	-35.96,77.20	104.02	24.90	-29.31,79.10	103.92
Interaction	1.89**	.62,3.16	29.37	1.79**	.58,3.00	27.82

All models include total brain volume, inverse distance to nearest major road, age, gender, race, education,

Income, overall health, BMI smoking status, and frequency of drinking alcohol. N = 18,278.

* p < .05.

** p < .01,

*** p < .001.

Source: *UK Biobank*.

**Table 6:** Thalamus volume (mm^3^) and interactions between air pollution and overall health: Unstandardized coefficient with 95% confidence intervals and standardized coefficients from linear regression.

	Left Thalamus Volume			Right Thalamus Volume		
	b	95% CI	Beta	b	95% CI	Beta
Particulate Matter 2.5						
Pollutant	-33.58	-73.78,6.63	-35.49	-31.47	-69.99,7.06	-33.26
Overall health	-58.87	-190.27,72.53	31.71	-51.88	-177.76,74.01	32.21
Interaction	9.07	-4.07,22.20	9.58	8.42	-4.17,21.00	8.90
Particulate Matter 2.5 to 10						
Pollutant	-57.87*	-104.21,-11.53	-52.12	-49.70*	-94.10,-5.29	-44.76
Overall health	-66.13	-163.53,31.26	32.39	-55.37	-148.69,37.95	32.79
Interaction	15.33*	.21,30.44	13.80	13.71	-.77,28.20	12.35
Particulate Matter 10						
Pollutant	-20.10	-42.23,2.03	-38.17	-15.14	-36.35,6.06	-28.76
Overall health	-54.58	-171.15,61.98	32.46	-35.43	-147.12,76.25	32.76
Interaction	5.36	-1.85,12.57	10.18	4.20	-2.71,11.11	7.98
Nitrogen Dioxide						
Pollutant	.39	-5.51,6.30	2.99	-1.02	-6.68,4.64	-7.73
Overall health	34.80	-17.24,86.85	31.32	25.40	-24.46,75.26	32.07
Interaction	-.13	-2.06,1.80	-.99	.25	-1.60,2.09	1.89
Nitrogen Oxides						
Pollutant	-.67	-3.49,2.15	-10.48	-1.47	-4.17,1.23	-22.83
Overall health	26.01	-16.44,68.46	31.40	17.85	-22.81,58.52	32.04
Interaction	.12	-.81,1.05	1.90	.32	-.57,1.21	5.00

All models include total brain volume, inverse distance to nearest major road, age, gender, race, education,

Income, overall health, BMI smoking status, and frequency of drinking alcohol. N = 18,278.

* p < .05.

** p < .01,

*** p < .001.

Source: *UK Biobank*.

Apart from an inverse association between PM _2.5–10_ and left thalamic volume, we did not find inverse associations between air pollution and decreased thalamic volumes in adjusted models. In the adjusted model evaluating the association between PM _2.5–10_ and left thalamic volume, every one-unit increase in PM _2.5–10_ was associated with a 0.15 percent decrease in left thalamic volume, or every 10-unit increase in PM _2.5–10_ was associated with a 1.54 percent decrease in left thalamic volume. For comparison, age-related declines in hippocampal volume are less than one percent per year for healthy young adults but 1.7 percent per year in older adults [35], similar to the 1.54 percent decrease in left thalamic volume we found for every ten-unit increase in PM _2.5–10_.

## Discussion

Previous studies of the association between thalamic volume and air pollution have been mixed. Power et al. [7] found an association between aggregate volume of the thalamus, caudate, pallidum, and putamen but did not investigate thalamic volume specifically, Casanova et al. [6] in their study investigating only PM_2.5_ found a positive association with thalamic volume. Our findings add to these studies by providing preliminary support for a possible association between air pollution and left thalamic volume. Although we found a significant association between PM_2.5–10_ and left thalamic volume but not with right thalamic volume, the coefficients in both cases were in the same direction (left, -12.06 and right, -8.71), raising the possibility that the analyses were underpowered to detect a significant association between PM_2.5–10_ and right thalamic volume. However, after accounting for the interactive effects of age, educational attainment, and self-rated health, all the air pollutants we included were associated with thalamic volume.

The interaction models suggested vulnerability in certain groups to air pollution effects on bilateral thalamic volume. Increasing age was associated with greater thalamic vulnerability to PM2_2.5–10_ and to PM_10_. Similarly, we also found an interaction between PM_2.5_, NO_2_, and NO_X_ and educational attainment such that a college degree appeared to be protective against PM_2.5_, NO_2_, and NO_X_ for both left and right thalamic volumes. There was also an interaction between self-rated health and PM_2.5–10_ such that better self-rated health appeared protective against the effects of PM_2.5–10_ on left thalamic volume. Therefore, one of the findings of this study is that although only PM2_2.5–10_ was associated with less left thalamic volume in the main-effects models, the interaction models suggested that some air pollutants were associated with decreases in both left and right volume of the thalamus in older participants (PM_2.5–10_, PM_10_) and in those with less than a college education (PM_2.5_. NO_2_, NO_X_).

In contrast to previous findings showing an association between PM_2.5_ and larger thalamic volume [6], we found no association between PM_2.5_ and either left nor right thalamic volume in the main-effect models. While reasons for this apparent discrepancy are unclear, they could be due to differences in study samples, or to differences in age, in that age was higher in the Casanova et al., 2016 study compared to ours. Further, Casanova et al. [6] included only women, whereas we included both women and men. We also analyzed left and right thalamic volumes separately. Clearly, these discrepant findings indicate the need for additional study on the effects of air pollution on thalamic volume.

It is unclear why the main effect of PM_2.5–10_ on thalamic volume loss in adjusted models demonstrated lower volume in the left but not right thalamus. However, we note that prior work of other sources of brain insult such as traumatic brain injury, which is typically associated with diffuse neuropathology, not only may affect the thalamus [12], but that the effect may be unilateral [13]. For example, Tate et al. [13] found an association between mild traumatic brain injury and decreased surface area of the left posterolateral thalamus, similar to our finding of an association between PM _2.5–10_ and only left but not right thalamic volume. Similarly, in prior work on air pollution also based on data from the UK Biobank, we found that less left but not right hippocampal volume was associated with higher pollution exposure in adjusted models [10]. We note that in both the current study and our prior study on the hippocampus, the coefficients were in the same direction for both left and right structures but were statistically significant on the left only. Perhaps this is a lateralized effect, perhaps there is less variability in volume on the left, or perhaps this is an issue related to power. Still, the interactions effects we found were generally bilateral.

While thalamic injury has been associated with cognitive function [14], whether the possible association between air pollution and thalamic volume might relate to cognitive and neuropsychiatric functions requires additional research. In a study also based on data from the UK Biobank, Cullen et al. [36] in adjusted models found only a weak association between air pollution and cognitive function. When interpreting these findings, though, it is important to consider that participants in the UK Biobank on average receive substantially less exposure to air pollution compared to people in some other regions of the world, where concentrations of air pollution can be much higher [28]. The preliminary evidence we found of an association between air pollution and thalamic volume particularly in some vulnerable groups indicates the need for additional research evaluating associations between air pollution and thalamic volume in regions where air pollution levels are higher.

Our findings suggesting a possible association between exposure to PM_2.5_, PM_2.5–10_, PM_10_, NO_2_, and NO_x_ and smaller thalamic volumes are consistent overall with previous studies that have reported associations between exposure to air pollution and cognitive dysfunction in both animal models and in humans. In a murine model, NO_2_ was associated with decreased cognitive function [37], and NO_2_ has been associated with cognitive dysfunction in humans [38]. A longitudinal study found an association between NO_2_ and hospitalization for dementia [39]. Similarly, the results of a longitudinal study in Sweden showed an association between NO_x_ and dementia incidence [40]. In older women, PM_2.5_ and PM_2.5–10_ were associated with faster cognitive decline [41].

While we did not investigate potential mechanism by which air pollution could affect thalamic volume, several factors suggest that it is biologically plausible that air pollution could affect thalamic structure and function. In this regard, animal models have shown associations between air pollution and neurodegeneration, brain proinflammatory cytokines [28], and neuronal loss [42], and mitochondrial dysfunction in a mouse model possibly could have mediated part of the association between cognitive dysfunction and NO_2_ [37]. Further, air pollution can result in neuroinflammation, oxidative stress [28], and prefrontal vascular damage [5], factors that could influence thalamic gray-matter volume. Because of associations between air pollution and prefrontal-cortical volume [5–7] and other cortical regions [9], it is possible that given the extensive connections between the thalamus and cortical areas, there might be diaschisis or Wallerian degeneration-related atrophy of the thalamus independent of or in addition to directly toxic effects of air pollution. Moreover, toxins in air pollution might reach the brain through the olfactory bulb or through the blood-brain barrier [3, 43]. While the exact mechanisms are largely unknown, even larger particles such as PM_10_ have been associated with neurologic disease. For example, studies have found an association between PM_10_ and multiple sclerosis including etiology [44] as well as with relapse [45]. PM_10_ has also been associated with ischemic stroke mortality [46]. PM_10_ has been shown to stimulate lung inflammation and endothelial injury [47] and encourage atherosclerosis [48]. In this regard, while we did not find associations between PM_2.5_ and either left or right thalamic volume, there was an association between PM_2.5–10_ and left thalamic volume, suggesting that larger particles can affect thalamic volume. Similarly, in the interaction models increasing age interacted with PM_2.5–10_ and PM_10_ resulting in smaller left and right thalamic volumes, whereas there was not an interaction with PM_2.5_ and age. In contrast, though, the interaction between PM_2.5_ and educational attainment was associated with smaller left and right thalamic volumes, whereas there were no such associations between educational attainment and either PM_2.5–10_ or PM_10._

The results from our interaction models suggest that the thalamus in older people and people with less education might be more vulnerable to air pollution than in younger people and people with more education. While we did not investigate mechanisms underlying these possible associations, one possibility might be related to the increased cognitive reserve in people with more education, which might decrease vulnerability to brain insult [49], including possibly air pollution. Likewise, the effects of air pollution on cognitive function appear to be stronger in older people [50].

Air pollution has been associated with dementia [1], indicating an association between exposure to air pollution and neurodegeneration, which possibly could include the thalamus. While findings showing associations between exposure to air pollution and neurotoxicity do not necessarily include the thalamus, the pathological processes such as neuroinflammation and oxidative stress associated with exposure to air pollution could extend to the thalamus.

In addition to the study’s cross-sectional design limiting conclusions about causality, several other factors could affect the interpretation of the study’s findings. Not all subjects in the UK Biobank had brain imaging, raising the possibility of selection bias. While the exposure variable–air pollution–was objective, it only covered one year of exposure; lifetime estimates of exposure to air pollution likely would be a better exposure variable. Studies investigating associations between exposure to air pollution and cognitive function and brain structure have relied on a variety of methods to determine exposure to air pollution [3], and the methods we relied on contain several potential limitations. In this regard, the magnetic resonance imaging data we used were obtained from 2014 to 2019, whereas the estimates for air pollution were obtained in 2010, leaving a gap between the attainment of the brain imaging and estimate of exposure to air pollution, a limitation that potentially could affect the associations we found. Similarly, the estimates of air pollution were for place of residence and did not take into account time spent away from home or occupational exposure to air pollution. We adjusted for several potentially confounding variables, but we might not have included other relevant variables, such as blood glucose or lipid concentrations, in our analyses, raising the possibility of residual confounding. One final possible limitation is that we did not control for multiple comparisons.

Although not necessarily a limitation, a further consideration when interpreting these findings is the strength of the exposure variable. In the UK Biobank sample, mean PM_2.5_ concentrations, for example, were slightly below the level of maximum exposure recommended by the World Health Organization [51], whereas many people across the world have much higher exposures [28].

## Conclusions

The results of this study taking into account its limitations suggest a possible association between exposure to air pollution and thalamic volume in adults, with some groups possibly being more susceptible to the effects of air pollution on thalamic volume. Increasing age and comparatively lower education might be risk factors for an effect of air pollution on the thalamus. Given the preliminary nature of these findings, they require verification particularly in samples where mean exposure to air pollution is higher than that in the UK Biobank. The effects of this association also require additional research to determine their effects if any on neurocognitive and neuropsychiatric function.
